# Seeing What’s on the Plate: Composition-Aware Fine-Grained Food Recognition for Dietary Analysis

**DOI:** 10.3390/foods15050931

**Published:** 2026-03-06

**Authors:** Linghui Ye, Qingbing Sang, Zhiyong Xiao

**Affiliations:** School of Artificial Intelligence and Computer Science, Jiangnan University, Wuxi 214122, China

**Keywords:** food recognition, fine-grained classification, Vision Transformer, key-region awareness, dietary analysis, feature fusion

## Abstract

Reliable visual characterization of food composition is a fundamental prerequisite for image-based dietary assessment and health-oriented food analysis. In fine-grained food recognition, models often suffer from large intra-class variation and small inter-class differences, where visually similar dishes exhibit subtle yet discriminative differences in ingredient compositions, spatial distribution, and structural organization, which are closely associated with different nutritional characteristics and health relevance. Capturing such composition-related visual structures in a non-invasive manner remains challenging. In this work, we propose a fine-grained food classification framework that enhances spatial relation modeling and key-region awareness to improve discriminative feature representation. The proposed approach strengthens sensitivity to composition-related visual cues while effectively suppressing background interference. A lightweight multi-branch fusion strategy is further introduced for the stable integration of heterogeneous features. Moreover, to support reliable classification under large intra-class variation, a token-aware subcenter-based classification head is designed. The proposed framework is evaluated on the public FoodX-251 and UEC Food-256 datasets, achieving accuracies of 82.28% and 82.64%, respectively. Beyond benchmark performance, the framework is designed to support practical image-based dietary analysis under real-world dining conditions, where variations in appearance, viewpoint, and background are common. By enabling stable recognition of the same food category across diverse acquisition conditions and accurate discrimination among visually similar dishes with different ingredient compositions, the proposed approach provides reliable food characterization for dietary interpretation, thereby supporting practical dietary monitoring and health-oriented food analysis applications.

## 1. Introduction

Dietary patterns have become increasingly diverse, accompanied by substantial visual variability in food presentation across regions, cooking styles, and consumption contexts. Such variability poses significant challenges for fine-grained food recognition, especially when visually similar food categories exhibit subtle yet discriminative differences in ingredient dominance and spatial organization, which in turn affect the reliability of image-based dietary assessment and food-related health analysis.

Traditional manual dietary recording is often labor-intensive and error-prone, limiting its scalability in practical monitoring scenarios. Consequently, recent studies have incorporated computer vision and deep learning techniques into dietary image analysis, enabling tasks such as automatic food category recognition, and in some cases, calorie-related analysis based on photographs captured in real-world dining environments [1]. These image-based approaches improve the efficiency and objectivity of dietary data acquisition [2], motivating further research on reliable fine-grained food recognition in health-related contexts. Against this background, fine-grained food recognition technology has gradually become a research hotspot in the field of intelligent nutrition analysis [3]. However, accurately distinguishing visually similar food categories under high intra-class variability remains a fundamental challenge for current visual recognition models [4].

Compared with traditional fine-grained tasks, food images pose greater challenges due to significant intra-class variations and inter-class visual overlaps caused by ingredient composition, cooking processes, and presentation styles. Specifically, food images often exhibit intra-class diversity [5], where the same dish may appear with noticeable differences in color, shape, and background due to variations in region, cooking style, or shooting angle [6]. Meanwhile, there also exists inter-class similarity, as certain stir-fried dishes or desserts made from similar ingredients or arranged in similar plating styles present highly comparable visual appearances [7], resulting in dispersed intra-class distributions and small inter-class distances in the feature space.

A number of large-scale datasets dedicated to food recognition have been successively introduced [8], such as Food-101 [9], UEC Food-256, and FoodX-251 [10]. The release of these datasets further highlights the complexity of real-world food recognition, where complex backgrounds, diverse plating patterns, and fine-grained visual ambiguity are commonly observed [11]. Developing models capable of accurately identifying and distinguishing fine-grained food categories is not merely a technical breakthrough, but also the key to improving the applicability, reliability, and trustworthiness of image-based dietary analysis systems [12].

Although mainstream models such as Swin-DR have achieved competitive performance, their architectures primarily rely on local window-based attention and multi-scale fusion, which limits explicit modeling of long-range spatial dependencies and discriminative region interactions [13], while significant limitations remain in spatial perception and key-region modeling [14].

**Figure 1:**
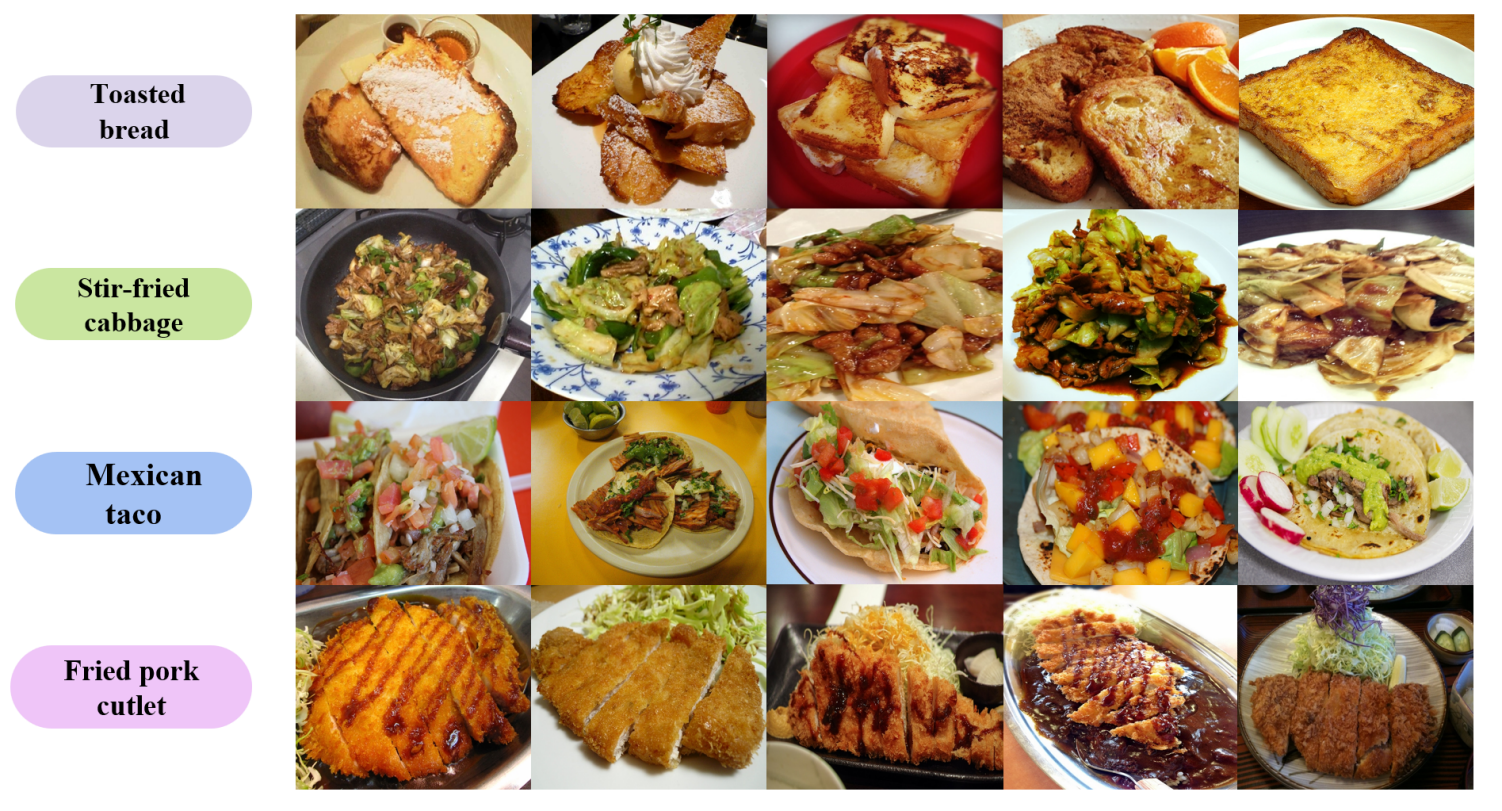
Representative samples from the FoodX-251 and UEC-Food256 datasets, showing significant intra-class variations and complex background conditions in fine-grained food recognition.

On the one hand, Swin Transformer has inherently limited spatial modeling capacity, making it difficult to effectively capture the complex variations in structure and arrangement within the same food category. Global layout differences are difficult to model adequately through local attention alone, thereby affecting classification stability and accuracy [15]. On the other hand, the model’s positional awareness and attention mechanisms are relatively weak [16], often leading to overfitting on background areas or irrelevant edges while lacking precise localization and emphasis on the main object. Particularly when dealing with mixed dishes such as boiled fish with chili or spicy hot pot, recognition becomes challenging to focus accurately on the key food regions. As a result, existing models struggle to jointly model global–local spatial relations, fine-grained intra-class variations, and background suppression [17]. As can be seen from Figure 1, food categories usually exhibit small inter-class differences, large intra-class variations, and high diversity in ingredients and plating forms. Therefore, models must not only finely model local structures but also maintain sensitivity to overall layout and positional relationships to break through existing performance bottlenecks.

Fine-grained food recognition requires the coordinated modeling of local textures, global spatial layouts, and salient ingredient regions; however, existing strategies often integrate these cues in a coarse manner, leading to suboptimal discriminative representation. In addition, conventional global aggregation and single-prototype classification schemes tend to discard spatial distribution cues and inadequately model pronounced intra-class variability. These issues collectively constrain further performance improvement in fine-grained food recognition. Hybrid CNN–Transformer architectures embed convolutional blocks into Transformer backbones to enhance joint modeling of local textures and global dependencies [18].

To address these challenges, we propose a unified fine-grained food recognition framework that emphasizes spatial structure modeling and composition-aware feature representation, aiming to improve the robustness and reliability of food recognition in complex real-world scenarios. From a food analysis perspective, explicitly modeling spatial structure and ingredient-dominated regions enables more stable characterization of composition patterns within a dish, which is essential to distinguish visually similar foods with different underlying ingredient arrangements. Enhancing positional awareness of key food regions allows the model to focus on composition-related visual cues, such as ingredient concentration areas, while effectively suppressing background elements unrelated to food structure. Existing feature integration strategies often fail to balance complementary visual cues effectively [19,20]. Accordingly, an adaptive feature integration strategy is required to preserve complementary information while suppressing redundancy and noise in fine-grained food recognition. Previous studies indicate that adaptive normalization and gating mechanisms can help balance heterogeneous feature contributions and improve robustness in complex visual recognition tasks [21]. Low-rank interaction and attention-based enhancement have also been shown to improve fine-grained discrimination while maintaining computational efficiency [22,23]. At the decision stage, conventional classification designs often suffer from spatial information loss and limited ability to model intra-class diversity, which constrains performance in fine-grained food recognition. Preserving spatial distribution cues during pooling has been shown to improve recognition of food categories with subtle structural and compositional differences [24]. Such spatially aware pooling strategies help maintain sensitivity to ingredient distribution and local structure in visually similar food categories [25].

Experimental results demonstrate the effectiveness of the proposed framework for fine-grained food image recognition under complex visual conditions. The proposed approach achieves accuracies of 82.64% on Food-256 and 82.28% on FoodX-251.

A single food category often exhibits substantial visual variation across regions and preparation styles, which increases the difficulty of consistent feature representation. Failure to capture variations in overall layout and local ingredient arrangements may lead to representation confusion, while insufficient positional awareness often causes attention to drift toward irrelevant regions, weakening focus on key ingredients.

The proposed framework integrates global layout modeling with enhanced key-region awareness to improve discrimination of subtle structural and compositional variations in fine-grained food images. By jointly modeling spatial structure and positional information, it strengthens sensitivity to ingredient-dominant regions while suppressing background interference, thereby improving discriminative representation under complex visual conditions. This approach provides reliable technical support for image-based dietary composition analysis and health-oriented food assessment pipelines.

The main contributions of this work are summarized as follows:

1. To handle complex spatial structures and subtle appearance variations caused by ingredient composition and preparation styles, we design AGRA (Adaptive Grouped Residual Attention) to jointly capture global layout and local ingredient arrangements in food images.
2. We introduce CEAG (Coordinate-Enhanced Adaptive Gating) to improve the model’s ability to localize key regions while suppressing background distractions such as plates and tablecloths, thus enhancing classification accuracy and robustness.
3. We propose SGLR-Mixer, a soft-gated low-rank fusion strategy that adaptively integrates heterogeneous visual cues in food images while avoiding redundant representations and excessive computational overhead.
4. We design TSCA-Classifier, which preserves spatial distribution cues during pooling and improves intra-class diversity modeling, enabling more reliable discrimination among visually confusing food categories.
5. We propose Swin-ACST, a fine-grained food classification framework that integrates spatial relationship modeling with key-region awareness, effectively enhancing feature discrimination and overall classification performance in complex food image analysis scenarios.

## 2. Related Works

### 2.1. Vision Transformers in Image Classification

In recent years, Vision Transformers (ViTs) have demonstrated remarkable modeling capabilities in image classification, emerging as a mainstream architecture following convolutional neural networks (CNNs). ViT was the first to directly apply the Transformer architecture to sequences of image patches [26,27], leveraging global self-attention to capture long-range dependencies. Subsequently, DeiT introduced efficient training strategies [28], improving data efficiency in Vision Transformer training.

To further reduce computational cost and enhance spatial modeling capability [29], Swin Transformer proposed a hierarchical architecture with a shifted-window mechanism [13], balancing global semantic modeling and local detail perception. Despite these advancements, Vision Transformers still face challenges in spatial structure understanding [30] and fine-grained modeling [31]. For instance, window partitioning limits cross-window spatial interaction, which may fragment global structural information. In fine-grained recognition scenarios, such fragmentation weakens sensitivity to subtle spatial configuration differences and ingredient-dominant regions, especially when foreground objects share similar textures but differ in layout organization. These limitations indicate that additional mechanisms are required to strengthen spatial structure modeling beyond standard window-based attention.

### 2.2. Spatial and Attention Mechanisms

Early works such as SENet enhanced representational capacity through channel attention [32], while CBAM further integrated spatial and channel attention to jointly model channel-wise and spatial importance [33].

In recent years, increasing research efforts have been devoted to the design of efficient spatial attention mechanisms, such as GENet, GALA, and Coordinate Attention. By incorporating spatial coordinate information [16], Coordinate Attention organically integrates positional encoding with channel attention [34], effectively improving the model’s spatial localization capability. Although these mechanisms improve regional emphasis, most of them operate either along channel dimensions or within limited spatial receptive fields, lacking explicit modeling of long-range spatial dependencies and global–local structural interactions. This limitation becomes more pronounced in fine-grained scenarios, where subtle differences often lie in ingredient arrangement rather than isolated local responses.

Therefore, spatial perception mechanisms should not only emphasize salient regions but also explicitly model positional relationships between ingredients to enhance discrimination in fine-grained food recognition tasks.

### 2.3. Global Context Modeling Techniques

Traditional convolutional networks, limited by their local receptive fields and inefficient long-range dependency capture, struggle to model global relationships effectively. To overcome these limitations, a variety of self-attention and non-local mechanisms, such as NLNet and GC-Net, have been proposed to strengthen the integration of global information within feature representations.

More recently, approaches like Grouped Residual Self-Attention have improved the efficiency of global–local feature interaction through grouped and residual structures [35], enhancing global spatial contextual sensitivity while balancing modeling capacity and computational cost.

In this work, we further optimize the perception of the global context, enhancing the model’s capability to capture subtle differences and improving classification performance in fine-grained food image recognition.

### 2.4. Image-Based Food Recognition for Dietary and Health-Oriented Analysis

Image-based food recognition has been widely studied as a fundamental component of dietary assessment and health-oriented food analysis [36], offering a non-invasive alternative to traditional manual dietary recording [37].

Compared with generic object recognition tasks, fine-grained food classification presents unique challenges due to large intra-class variation and small inter-class differences. Dishes belonging to the same category may differ substantially in appearance because of variations in ingredient dominance, cooking style, and presentation, while visually similar foods prepared with comparable ingredients often exhibit subtle structural differences. These characteristics make accurate and stable classification particularly important for reliable food characterization.

Recent research has increasingly emphasized the role of spatial structure modeling and region-aware feature representation in improving classification consistency for food images [38]. Nevertheless, many existing approaches primarily rely on backbone enhancement or data augmentation strategies, without explicitly modeling structured spatial configuration patterns that characterize ingredient composition and layout diversity. As a result, discriminative capacity remains limited when visually similar categories differ mainly in spatial arrangement rather than texture appearance.

## 3. Methods

### 3.1. Overall Architecture of the Approach

**Figure 2:**
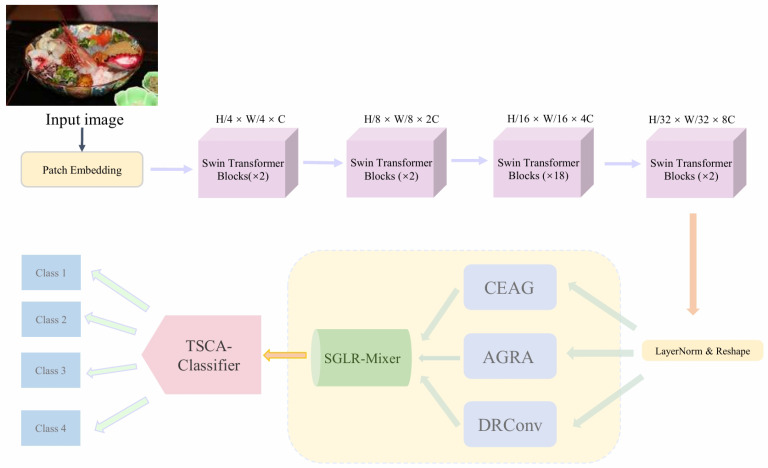
Overall architecture of the proposed Swin-ACST framework, illustrating the collaborative relationships among feature extraction, fusion, and classification stages.

The overall architecture of the proposed model is shown in Figure 2. The model mainly consists of three parts: the first part is the backbone network, the second part is the spatial perception and modeling enhancement module, and the third part is the fine-grained food classifier.

Among them, the backbone network is composed of multiple Swin Transformer modules. The spatial perception and modeling enhancement module sequentially integrates global spatial relation modeling enhancement and coordinate-aware spatial enhancement, combined with a DRConvBlock to further improve local feature representation ability. After multi-branch enhancement, to robustly integrate heterogeneous semantic information, SGLR-Mixer is introduced to adaptively integrate heterogeneous features from multiple enhancement paths, enabling stable fusion of complementary spatial and semantic information. Then, spatial selective amplification is applied to further strengthen the response of salient regions and suppress background interference.

The fine-grained food classifier no longer adopts a simple global average and single-center metric, but employs a combination of weighted aggregation-preserving spatial distribution and multi-center angular metric, namely the TSCA-Classifier, to preserve the spatial distribution of key food components and improve the modeling of intra-class appearance diversity.

The overall process is as follows: First, the three-channel RGB fine-grained food image is input into the backbone network, and the Swin Transformer extracts global feature representations. Then, the feature information is processed by AGRA for global and local relation modeling, which focuses on both overall structure and local arrangement to enhance spatial sensitivity and subtle-difference discrimination ability. Next, the CEAG module, based on coordinate awareness and spatial gating, improves the model’s attention accuracy to target regions, effectively suppresses irrelevant background areas, and avoids background overfitting. After that, local feature enhancement is performed to improve edge and texture representations.

For the features obtained from the above multi-branch enhancement, SGLR-Mixer performs path alignment and soft-gated low-rank fusion, highlighting salient regions and producing stable and more discriminative global representations. Finally, the enhanced features are fed into the TSCA-Classifier, where weighted aggregation and a multi-center angular metric are used to complete category prediction.

Overall, through multi-level spatial relation modeling, salient region localization, and robust fusion, the proposed model effectively enhances spatially aware feature representation and category discrimination for fine-grained food images, providing more stable and reliable predictions under diverse presentation conditions.

### 3.2. CEAG Block

Following the Swin-DR backbone, this section details the proposed CEAG Block to improve spatial awareness in fine-grained food image analysis. In food images, discriminative visual cues are often closely tied to the spatial distribution of ingredients and their relative positions within a dish, while irrelevant background elements such as plates or tablecloths may introduce significant interference. However, the window-based attention mechanism of Swin Transformer exhibits limited explicit positional sensitivity, making it difficult to consistently emphasize key food regions. The CEAG block addresses this issue by incorporating coordinate-aware attention to explicitly encode spatial location information into channel representations. Compared with conventional channel attention mechanisms that aggregate global spatial information into a single descriptor, Coordinate Attention preserves directional positional encoding along horizontal and vertical axes. This property makes it particularly suitable for food images, where ingredient distribution often follows structured spatial patterns rather than uniform global appearance statistics. This design enables the model to better align visual responses with ingredient-dominated regions while suppressing non-food background areas. By enhancing spatial localization and region-level discrimination, CEAG provides more reliable feature representations for fine-grained food recognition and downstream food composition analysis. The CEAG Block comprises two components: Coordinate Attention and spatial gating. Coordinate Attention encodes spatial locations and channel dependencies to provide strong coordinate guidance. It uses horizontal and vertical global average pooling to produce spatial embeddings:

(1) $$z_{c}^{h}=\frac{1}{W}\sum_{i=1}^{W}x_{c}\left(h,i\right)$$

(2) $$z_{c}^{w}=\frac{1}{H}\sum_{j=1}^{H}x_{c}\left(j,w\right)$$

These embeddings are concatenated and processed via 1×1 convolutions, BatchNorm2d, and h-swish to yield fused spatial features f:

(3) $$f=\mathrm{h-swish}\left(\mathrm{BN}2d\left(\mathrm{Conv}2d1\times 1\left([z_{c}^{h},z_{c}^{w}]\right)\right)\right)$$

Here, BN2d denotes 2D batch normalization, and h-swish is the hard-swish activation function. f is then split into horizontal and vertical attention weights via separate 1×1 convolutions and sigmoid activation:

(4) $$g^{h}=\sigma \left(\mathrm{Conv}2d{1\times 1}^{h}\left(f\right)\right),\;g^{w}=\sigma \left(\mathrm{Conv}2d{1\times 1}^{w}\left(f\right)\right)$$

Input features X are recalibrated in space and across channels to produce $X^{\prime }$:

(5) $$X^{\prime }=X⊙g^{h}⊙g^{w}$$

where ⊙ is element-wise multiplication. This encodes spatial coordinates explicitly, improving localization and suppressing background. The CEAG Block uses a dual gating mechanism. The first gate uses 1×1 convolution and sigmoid to produce feature gate G, and modulates the recalibrated features via a residual path:

(6) $$G=\sigma \left(\mathrm{Conv}2d1\times 1(X^{\prime })\right)$$

(7) $$Y=X^{\prime }+\alpha \cdot \left(X^{\prime }⊙G\right)$$

Here, α is a scaling factor (set to 0.1), and $G\in R^{C\times H\times W}$ is the feature gate. This gate focuses on spatially relevant regions and reduces background contributions. The second gate produces spatial gate M via 1×1 convolution and sigmoid, modulating the coordinate-enhanced features. Results are merged with the input via a residual path to yield output O:

(8) $$M=\sigma \left(\mathrm{Conv}2d1\times 1(Y)\right)$$

(9) $$O=Y+\beta \cdot \left(Y⊙M\right)$$

Here, β is a scaling factor (set to 0.1), and $M\in R^{1\times H\times W}$ is the spatial gate. The dual gating refines features by focusing on relevant spatial regions and suppressing background to reduce overfitting. BatchNorm2d and nonlinearities (e.g., h-swish or ReLU) are applied after each convolution for stable, expressive distributions. The design enables progressive refinement: Coordinate Attention provides spatial awareness, and the dual gates refine representations. By integrating Coordinate Attention and dual gating into a single block, CEAG improves focus on relevant regions, suppresses background, reduces overfitting, and enhances recognition on complex scenes and fine-grained targets.

### 3.3. AGRA Block

The Swin Transformer employs window-based local self-attention, which limits its capacity for comprehensive spatial modeling. To solve it, we design a novel Adaptive Grouped Residual Attention (AGRA) module with lightweight adaptive weights. The core innovation of AGRA lies in its ability to simultaneously capture both global structural information and local spatial arrangements, thereby significantly enhancing the model’s sensitivity to fine-grained spatial details. By explicitly modeling relationships between distant and nearby spatial positions, AGRA enables the network to better distinguish subtle structural differences arising from ingredient distribution and arrangement, which is crucial for fine-grained food recognition.

Given the input feature $X\in R^{B\times N\times C}$, the module first splits the channel dimension into two equal groups, denoted as $X_{1},X_{2}\in R^{B\times N\times \frac{C}{2}}$. For each group, it applies a grouped residual linear transformation to generate the query, key, and value representations, while introducing learnable adaptive weights $\alpha_{1},\alpha_{2}$ to control the contribution of the residual branch. Specifically, the transformation for each group is formulated as

(10) $$Q_{1}=X_{1}+\alpha_{1}\cdot {\mathrm{Linear}}_{Q}\left(X_{1}\right),\;Q_{2}=X_{2}+\alpha_{2}\cdot {\mathrm{Linear}}_{Q}\left(X_{2}\right),$$

(11) $$K_{1}=X_{1}+\alpha_{1}\cdot {\mathrm{Linear}}_{K}\left(X_{1}\right),\;K_{2}=X_{2}+\alpha_{2}\cdot {\mathrm{Linear}}_{K}\left(X_{2}\right),$$

(12) $$V_{1}=X_{1}+\alpha_{1}\cdot {\mathrm{Linear}}_{V}\left(X_{1}\right),\;V_{2}=X_{2}+\alpha_{2}\cdot {\mathrm{Linear}}_{V}\left(X_{2}\right).$$

The grouped *Q*, *K*, and *V* are then concatenated and reshaped for multi-head attention computation. *Q* and *K* are then normalized, and the cosine similarity, scaled by a learnable parameter λ, is computed to obtain the attention map. The attention calculation is given by

(13) $$\mathrm{Attn}=\mathrm{Softmax}\left(\lambda \cdot \frac{Q}{∥Q∥}\cdot {\left(\frac{K}{∥K∥}\right)}^{⊤}+B_{\mathrm{ES-RPB}}\right).$$

Here, λ is a learnable scaling parameter, ∥·∥ denotes the L2 norm, ⊤ denotes matrix transpose, and $B_{\mathrm{ES-RPB}}$ denotes the Exponential-Space Relative-Position Bias. To further enhance the model’s ability to capture spatial relationships and suppress background noise, it employs exponential mapping for the relative-position coordinates. Compared with conventional relative-position bias that relies on discretized positional indices, the exponential mapping introduces a continuous and smoothly bounded transformation of relative coordinates. For any two tokens with relative coordinates (ΔX,ΔY), it computes

(14) $$\Delta \hat{X}=\mathrm{sign}\left(\Delta X\right)\cdot (1-\exp(-|\Delta X|)),$$

(15) $$\Delta \hat{Y}=\mathrm{sign}\left(\Delta Y\right)\cdot (1-\exp(-|\Delta Y|)),$$

(16) $$B_{\mathrm{ES-RPB}}=\mathrm{MLP}\left(\left[\Delta \hat{X},\Delta \hat{Y}\right]\right).$$

The output of the attention is then split into two groups, and each group is projected back to the original feature space using a grouped residual linear layer with the same adaptive weights:

(17) $$\left[A_{1},A_{2}\right]=\mathrm{Split}\left(\mathrm{Attn}@V\right),$$

(18) $$O_{1}=X_{1}+\alpha_{1}\cdot {\mathrm{Linear}}_{\mathrm{proj}}\left(A_{1}\right),\;O_{2}=X_{2}+\alpha_{2}\cdot {\mathrm{Linear}}_{\mathrm{proj}}\left(A_{2}\right),$$

(19) $$O=\mathrm{Concat}(O_{1},O_{2}).$$

This design enables the adaptive control of information flow across grouped attention branches, thereby enhancing the robustness and discriminative capability of spatial attention modeling. The introduction of exponential-space relative-position bias further strengthens the modeling of spatial relevance across different structural regions, facilitating more consistent discrimination of fine-grained food patterns with complex spatial arrangements. The attention operation retains the quadratic complexity of standard self-attention with respect to token number, and the grouped projections do not alter its asymptotic complexity.

### 3.4. SGLR-Mixer

**Figure 3:**
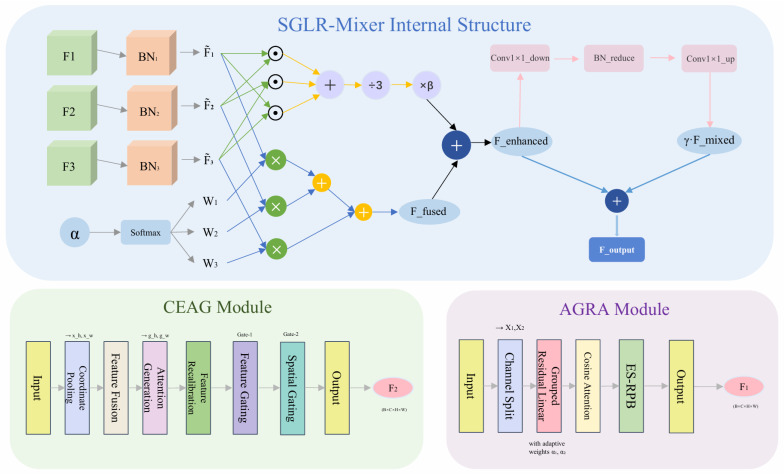
Structural illustration of the proposed AGRA, CEAG, and SGLR-Mixer modules, highlighting their internal mechanisms and information flow.

In fine-grained food classification, multi-branch feature extraction is commonly adopted to capture complementary cues related to global layout, ingredient-dominant regions, and local structural details. However, in food images with complex composition and high visual variability, simple fusion strategies such as direct concatenation or uniform weighting often introduce feature redundancy, noise accumulation, and unnecessary computational overhead, which may lead to unstable category predictions under real-world conditions. To address this issue, we propose a Soft-Gated Low-Rank Mixer (SGLR-Mixer) that performs adaptive fusion of three heterogeneous feature streams in a lightweight and structured manner. By selectively integrating spatial structure-aware features and key-region-focused representations, SGLR-Mixer enhances the consistency and reliability of the final classification output, which is critical for composition-aware food recognition serving downstream dietary analysis applications. The fusion module relies on element-wise operations and low-rank 1 × 1 convolutions, resulting in linear complexity with respect to spatial resolution and reduced quadratic complexity with respect to channel dimension. The framework diagram of SGLR-Mixer and its role within the overall multi-branch enhancement pipeline are illustrated in Figure 3.

Given three inputs $F_{1},F_{2},F_{3}\in R^{B\times C\times H\times W}$, we apply batch norm temporally to align magnitude differences:

(20) $${\tilde{F}}_{1}={\mathrm{BN}}_{1}\left(F_{1}\right),\;{\tilde{F}}_{2}={\mathrm{BN}}_{2}\left(F_{2}\right),\;{\tilde{F}}_{3}={\mathrm{BN}}_{3}\left(F_{3}\right)$$

Then, we fuse the following via learnable soft-gated weights $\alpha =[\alpha_{1},\alpha_{2},\alpha_{3}]$:

(21) $$w=\mathrm{Softmax}\left(\alpha \right)=\left[w_{1},w_{2},w_{3}\right]$$

(22) $$F_{\mathrm{fused}}=w_{1}\cdot {\tilde{F}}_{1}+w_{2}\cdot {\tilde{F}}_{2}+w_{3}\cdot {\tilde{F}}_{3}$$

To capture complementary information, we add a small second-order interaction term:

(23) $$F_{\mathrm{enhanced}}=F_{\mathrm{fused}}+\beta \cdot \frac{{\tilde{F}}_{1}⊙{\tilde{F}}_{2}+{\tilde{F}}_{2}⊙{\tilde{F}}_{3}+{\tilde{F}}_{1}⊙{\tilde{F}}_{3}}{3}$$

where β is the interaction coefficient and ⊙ is element-wise multiplication. A low-rank channel mixer performs lightweight cross-modal interaction. The channels are reduced to a low-rank space:

(24) $$F_{\mathrm{reduced}}={\mathrm{BN}}_{\mathrm{reduce}}\left(\mathrm{Conv}{1\times 1}^{\mathrm{down}}\left(F_{\mathrm{enhanced}}\right)\right)$$

and then mapped back to the original channel space:

(25) $$F_{\mathrm{mixed}}=\mathrm{Conv}{1\times 1}^{\mathrm{up}}\left(F_{\mathrm{reduced}}\right)$$

Finally, the mixed features are added back to the enhanced features via a residual:

(26) $$F_{\mathrm{output}}=F_{\mathrm{enhanced}}+\gamma \cdot F_{\mathrm{mixed}}$$

where γ is a scaling factor controlling the residual contribution.

### 3.5. TSCA-Classifier

**Figure 4:**
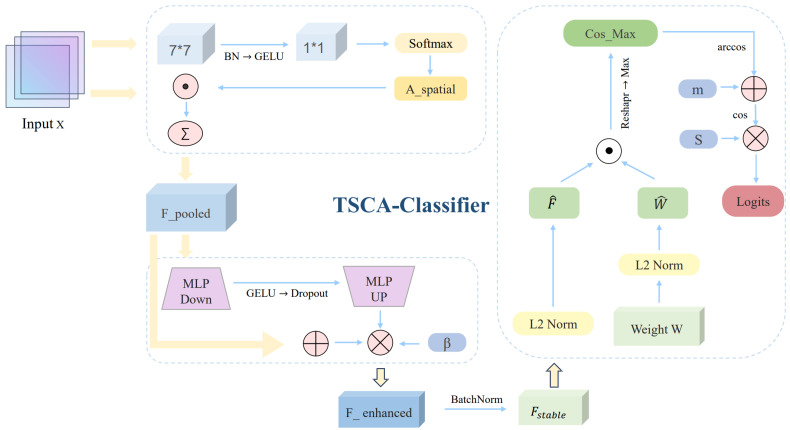
Architecture of the TSCA-Classifier, demonstrating its spatially weighted pooling and multi-center angular discrimination strategy.

In fine-grained food classification, global average pooling and single-centroid classifiers tend to discard spatial distribution cues and inadequately model intra-class diversity, which are critical for distinguishing visually similar food categories with different composition-related characteristics. To address this limitation, the TSCA-Classifier is proposed, as illustrated in Figure 4.

Given inputs $X\in R^{B\times C\times H\times W}$, the classifier uses Token-aware Pooling (TAP) to aggregate spatial features. TAP uses two-layer convolutions to generate spatial attention:

(27) $$A_{\mathrm{spatial}}=\mathrm{Softmax}\left(\mathrm{Conv}1\times 1\left(\mathrm{GELU}\left(\mathrm{BN}\left(\mathrm{Conv}7\times 7\left(X\right)\right)\right)\right)\right),$$

(28) $$F_{\mathrm{pooled}}=\sum_{i=1}^{H\times W}A_{\mathrm{spatial}}^{\left(i\right)}\cdot X^{\left(i\right)}$$

with 7×7 to C/16 and 1×1 to single-channel. A lightweight enhancement follows:

(29) $$F_{\mathrm{enhanced}}=F_{\mathrm{pooled}}+\beta \cdot \mathrm{MLP}\left(\mathrm{Dropout}\left(\mathrm{GELU}\left(\mathrm{MLP}\left(F_{\mathrm{pooled}}\right)\right)\right)\right)$$

where β=0.008; MLP maps C→C/32→C; Dropout =0.7. BatchNorm is then applied:

(30) $$F_{\mathrm{stable}}=\mathrm{BN}\left(F_{\mathrm{enhanced}}\right)$$

SubCenter ArcFace keeps *K* sub-centers per class (K=3), weight matrix $W\in R^{\left(C\times K\right)\times N_{\mathrm{classes}}}$, and $N_{\mathrm{classes}}$ is the number of classes. The features and weights are normalized as follows:

(31) $$\hat{F}=\frac{F_{\mathrm{stable}}}{\left|\right|F_{\mathrm{stable}}\left|\right|2},\;\hat{W}=\frac{W}{||W||2}$$

Cosine similarity:

(32) $$\cos=\hat{F}\cdot {\hat{W}}^{T}$$

Reshape to $\left[B,N_{\mathrm{classes}},K\right]$ and take the max over *K* sub-centers:

(33) $$\cos_{\max}=\max_{j=1}^{K}\cos_{i,j}$$

Train with an angular margin *m*:

(34) $$\theta =\arccos(\cos_{\max})$$

The target logit for the ground-truth class is

(35) $$\cos_{\mathrm{target}}=\cos\left(\theta +m\right)$$

Final logits are obtained by scaling with *s*:

(36) $$\mathrm{logits}=s\cdot \cos_{\mathrm{final}}$$

Here, arccos and cos denote the inverse cosine and cosine functions, respectively, *s* is a scaling factor (set to 16.0), and *m* is the angular margin (set to 0.12).

## 4. Experiments and Results

### 4.1. Dataset

This paper employs two common fine-grained food image datasets to evaluate the proposed method. All datasets adhere to official standard partitions.

- **FoodX-251.** FoodX-251 comprises 251 visually similar fine-grained categories (e.g., cakes with varying decorations [10], sandwiches with distinct fillings, and pasta in diverse shapes). The dataset uses 118 k images as a training set and 40 k images for validation and testing.
- **UEC FOOD-256.** UEC FOOD 256 includes 256 categories of food images [39], each annotated with bounding boxes precisely localizing food regions. The dataset primarily features Japanese cuisine (e.g., tamagoyaki and takoyaki) alongside international dishes, where certain Japan-specific categories may present recognition challenges for non-native observers.

### 4.2. Evaluation Metrics

To comprehensively assess the effectiveness of our proposed method in image classification, three widely accepted evaluation metrics were employed: Top-1 Accuracy, F1 Score, and Precision.

- Accuracy measures the proportion of correctly predicted samples over the total number of test instances, indicating overall performance.
- Precision reflects how many of the predicted positive results are actually correct.
- Recall represents the ability of the model to correctly identify all actual positive instances.
- F1 score is the harmonic mean of precision and recall, providing a balanced metric between the two.

The definitions of these metrics are given as

(37) $$\mathrm{Acc}=\frac{1}{N}\sum_{i=0}^{N-1}\left(f\left(x_{i}\right)=y_{i}\right)$$

(38) $$\mathrm{Recall}=\frac{TP}{TP+FN}$$

(39) $$\mathrm{Precision}=\frac{TP}{TP+FP}$$

(40) $$F1=\frac{2\cdot \mathrm{Precision}\cdot \mathrm{Recall}}{\mathrm{Precision}+\mathrm{Recall}}$$

Here, TP (True Positives) refers to correctly classified positive samples, FP (False Positives) are negative samples mistakenly predicted as positive, FN (False Negatives) are positive samples incorrectly classified as negative, and TN (True Negatives) are correctly classified negative samples.

### 4.3. Performance Comparative Experiments

To evaluate the effectiveness of the proposed method in fine-grained food recognition tasks, systematic comparative experiments were conducted on two authoritative public datasets, FoodX-251 and UEC FOOD-256. The compared methods include representative image recognition architectures, covering both conventional convolutional neural networks (e.g., ResNet, TResNet, EfficientNet) and a variety of recently developed Vision Transformer-based models (e.g., ViT, Swin Transformer, Twins, CaiT, ConvNeXt, CSwin, VOLO) [40,41].

All experiments were performed on a single NVIDIA GeForce RTX 4090 GPU with mixed-precision training to improve computational efficiency. The batch size was set to 8, and the AdamW optimizer was employed. A cosine learning-rate schedule was adopted with 5 warmup epochs starting from a base learning rate of 5.00 × $10^{-4}$ and a weight decay of 0.03, and each model is trained for 50 epochs. Data preprocessing and augmentation included RandomResizedCrop, RandomHorizontalFlip, TrivialAugmentWide, RandomErasing, and standard ImageNet normalization. Swin-ACST was initialized with ImageNet-22K pre-trained weights of Swin Transformer. All experiments were conducted with a fixed random seed of 42. To ensure fairness and reproducibility, three commonly used evaluation metrics, Top-1 Accuracy, F1 Score, and Precision, were adopted to comprehensively assess the classification capability of different models under multi-class and imbalanced food image distributions.

As shown in Table 1, the performance differences among the compared models are evident across both datasets. Traditional convolutional networks exhibit certain advantages in local texture modeling, yet they often fail to establish global semantic consistency when facing food images with complex backgrounds or diverse structural forms. Transformer-based architectures, by contrast, improve the integration of global features through self-attention mechanisms, thereby achieving higher recognition accuracy. However, these methods still face limitations when handling food categories with large intra-class variations and scattered discriminative features.

Our proposed model incorporates several optimization mechanisms tailored for fine-grained food imagery, effectively enhancing its capabilities in spatial modeling, region perception, and detail discrimination. On the FoodX-251 and UEC FOOD-256 datasets, our method achieves 82.28% and 82.64% Top-1 accuracy, respectively, confirming its superiority and robustness in complex food recognition scenarios.

The model centers around unified spatial relation modeling and composition-related salient region localization, strengthening spatial dependency representations across both global and local levels. Through robust alignment of heterogeneous semantic branches and lightweight low-rank fusion, the proposed architecture enables effective feature integration that preserves the spatial distribution of key regions while suppressing background noise and redundancy. Based on this, the classification head replaces the single-center paradigm with a multi-center angular metric, enhancing intra-class diversity representation and boundary robustness. With only marginal additional computational overhead, the entire pipeline achieves end-to-end optimization from feature extraction and cross-branch fusion to decision-space refinement, significantly improving recognition stability and discriminative capability in “same-class samples with different composition layouts” and cluttered background scenarios.

Notably, the proposed method demonstrates strong discriminative capability in handling visually similar food categories that belong to different semantic classes, a scenario that frequently occurs in real-world food image analysis. For example, soup-based dishes such as beef soup and pork bone soup often present highly comparable visual appearances in terms of color tone, liquid dominance, and serving context, despite differing in primary ingredient and dietary relevance. Conventional models tend to confuse such categories due to their reliance on coarse global representations. In contrast, the proposed framework can accurately attend to composition-related regions while modeling global spatial relationships, enabling more reliable differentiation between visually confusing yet semantically distinct food categories and maintaining stable classification performance under complex background conditions.

These results demonstrate that the proposed approach achieves systematic improvements in feature alignment, category boundary modeling, and local–global information fusion by integrating more efficient spatial dependency modeling and region-aware mechanisms. Consequently, it enhances the model’s ability to discriminate subtle inter-class differences and greatly improves its classification robustness and stability in fine-grained food recognition.

### 4.4. Ablation Analysis

To verify the effectiveness of the overall architectural design proposed in this study, we conducted a series of ablation experiments on two fine-grained food image datasets, FoodX-251 and UEC FOOD-256. Starting from the baseline backbone, the experiments follow the overarching principle of “enhancing spatial relation modeling—emphasizing key regions—robust multi-branch fusion and discrimination”. Each capability is introduced progressively to assess its individual and cumulative contribution to the overall recognition performance, while avoiding isolated or fragmented evaluations of single modules.

Specifically, the original Swin-DR model without any structural enhancement is designated as the baseline. As shown in Table 2, its Top-1 accuracies on the two datasets are 81.07% and 82.15%, respectively. After incorporating spatial relation modeling enhancement, the accuracies increase to 81.41% and 82.36%, indicating that such modeling facilitates better representation of global layouts and cross-region dependencies, yielding stable gains particularly on the UEC FOOD-256 dataset. Further introducing discriminative region focusing and guidance leads to accuracies of 81.67% and 82.48%. When these capabilities are jointly integrated under a unified framework with lightweight low-rank multi-branch fusion, the model achieves 81.97% and 82.54%, respectively. Finally, after convergence through a more robust multi-center discriminative space, the model attains its best performance of 82.28% on FoodX-251 and 82.64% on UEC FOOD-256. These results are clearly superior to those achieved by any single modification, validating the synergistic effect of the overall design in spatial representation, salient localization, and heterogeneous feature integration.

Taking visually similar soup-based dishes as an example, categories such as beef soup and chicken soup often exhibit highly comparable appearances, characterized by dominant liquid regions, similar color distributions, and overlapping serving contexts. Differences in primary ingredients are frequently reflected only in subtle local regions and spatial composition cues, making them prone to confusion under background interference and plating variations. The baseline model tends to be distracted by dominant background or container regions, resulting in unstable attention and inconsistent predictions. In contrast, when the proposed integrated strategy is enabled, the model can consistently emphasize ingredient-related regions within the global layout, preserve meaningful spatial distribution patterns, and suppress irrelevant background responses, thereby achieving more reliable discrimination between visually confusing yet semantically distinct food categories.

In summary, the ablation results indicate that the observed performance gains are not attributable to any single component in isolation, but arise from the coordinated optimization of spatial relation modeling, salient region localization, robust low-rank fusion, and discriminative decision modeling. This integrated design enhances the consistency of feature representation, stabilizes region-aware responses, and improves fine-grained alignment across complex visual conditions. These findings further validate the structural rationality of the proposed framework and its practical suitability for reliable fine-grained food image recognition in application-oriented settings.

## 5. Dietary Analysis Application

In recent years, increasing attention has been paid to dietary health and nutritional balance. However, in daily dining scenarios, many consumers lack accurate knowledge of food composition and ingredient structure, especially when visually similar dishes differ subtly in their main ingredients or preparation styles. This often leads to misunderstandings in dietary assessment and limits the effectiveness of image-based dietary analysis.

To address this problem, we implement a prototype image-based dietary analysis application in which the proposed Swin-ACST framework serves as the core food recognition module. The application is designed to support reliable food category identification under real-world dining conditions and to provide users with consistent food-related information for subsequent dietary interpretation. It should be noted that the present application focuses exclusively on food category recognition as a foundational component of dietary analysis systems.

**Figure 5:**
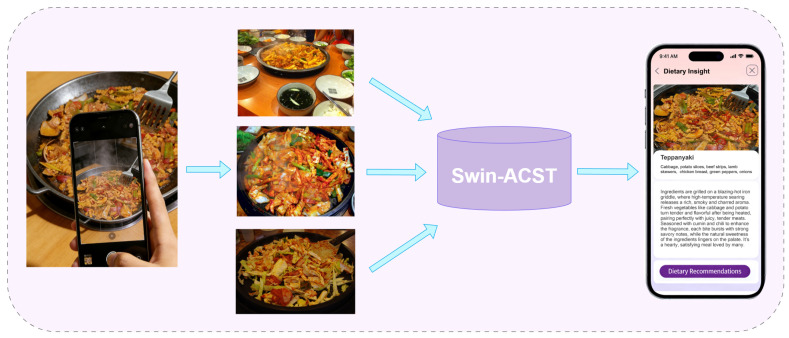
Despite significant variations in visual appearance caused by real-world dining environments, the system consistently identifies the food as the same category, demonstrating stable recognition performance and robustness for practical dietary monitoring.

As illustrated in Figure 5, users capture food images using mobile devices in unconstrained environments, where variations in shooting angle, illumination, distance, and background are common. The captured images are uploaded through the application interface and transmitted to the inference server, where the trained Swin-ACST model performs fine-grained food recognition. Despite substantial visual variations in image appearance, the system maintains relatively consistent recognition results for the same food category across different capture conditions. This indicates that the proposed framework effectively captures food-related structural and compositional cues that remain reliable across different acquisition conditions, which is essential for practical dietary monitoring.

**Figure 6:**
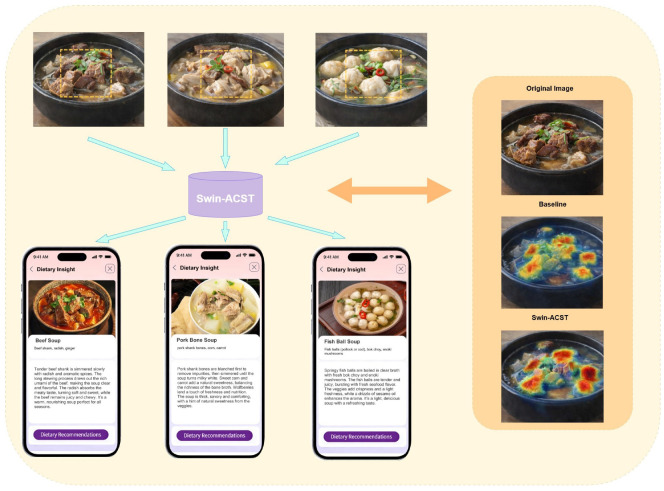
For food images with highly similar appearance, the proposed model highlights discriminative ingredient regions while suppressing irrelevant background areas. The system is able to focus on category-specific visual cues, enabling accurate differentiation between food items that are easily confused in real-world dietary scenarios.

Beyond robustness to appearance variation, accurate discrimination among visually similar food categories is particularly important for dietary analysis. As shown in Figure 6, several dishes with highly similar visual appearances but different ingredient composition are evaluated. The visualization results demonstrate that the proposed system focuses on ingredient-dominant regions while suppressing irrelevant background areas, enabling more precise differentiation between visually confusing dishes. Such behavior is critical in dietary applications, where misclassification between similar-looking foods may lead to incorrect interpretation of nutritional characteristics. Nevertheless, certain limitations should be acknowledged. Recognition performance may be affected under conditions such as severe occlusion, extreme illumination variation, complex multi-dish scenes, or domain shifts not represented in the training datasets. These factors constitute potential sources of error in practical deployment.

After food recognition is completed, the system automatically retrieves corresponding food-related information and health-oriented references from the database according to the recognized category and returns the results to the application interface. By reliably distinguishing food categories with subtle compositional and structural differences, the application supports more meaningful interpretation of meal composition and provides users with clearer dietary insights. These results indicate that Swin-ACST is well suited for deployment in real-world image-based dietary analysis applications, providing a reliable visual foundation for intelligent food monitoring and health-oriented dietary assessment.

## 6. Conclusions

This study presents Swin-ACST, a fine-grained food classification framework designed to enhance the visual characterization of composition-related structures in food images. By jointly modeling global spatial layouts and local structural arrangements, the proposed approach effectively addresses key challenges in fine-grained food recognition, including large intra-class variation and small inter-class differences commonly observed in visually similar dishes.

The introduced spatial relation modeling and key-region awareness mechanisms enable the model to suppress background interference and maintain consistent attention to ingredient-dominant regions, improving both inter-class discrimination and intra-class consistency. In addition, the adaptive multi-branch fusion strategy and token-aware subcenter-based classification head further strengthen class separability by preserving spatial information and explicitly modeling intra-class diversity. Experimental results on FoodX-251 and UEC Food-256 demonstrate that Swin-ACST achieves superior performance compared with existing advanced methods, particularly for visually confusing food categories. Despite these improvements, several limitations should be acknowledged. The proposed architecture introduces additional structural components, which increase model complexity compared to lightweight baselines. Furthermore, cross-domain generalization to more diverse food datasets or real-world deployment environments requires further investigation.

Beyond classification accuracy, the proposed framework provides a robust and non-invasive visual characterization basis for composition-aware food recognition in health-oriented applications. By reliably distinguishing food categories that differ subtly in ingredient dominance and spatial organization, Swin-ACST supports more consistent interpretation of meal composition and its potential dietary relevance. This work demonstrates that enhancing spatial structure modeling and key-region awareness is an effective strategy for bridging fine-grained food recognition with practical dietary analysis scenarios, offering a reliable visual foundation for intelligent food analysis systems.

## Figures and Tables

**Table 1:** Swin-ACST compares results with other models on the FoodX-251 and UEC Food-256 datasets. The short horizontal line represents the indicators that were not evaluated in the cited article.

Methods	Epochs	Resolution	FoodX251			UECFood-256		
			Acc. (%)	Pre. (%)	F1. (%)	Acc. (%)	Pre. (%)	F1. (%)
ResNet-50	50	224 × 224	72.13	72.05	72.93	75.54	75.44	76.06
ResNet-101	50	224 × 224	73.11	73.10	74.00	75.65	75.57	76.30
TResNet-L	50	224 × 224	74.84	74.82	75.61	76.18	76.01	76.74
TResNet-XL	50	224 × 224	74.59	74.51	75.27	76.35	76.17	76.77
EfficientNet-b7	50	224 × 224	73.54	73.44	74.15	74.84	74.52	75.05
ConvNext-B	50	224 × 224	77.84	77.75	78.35	78.79	78.60	79.11
ConvNext-L	50	224 × 224	78.02	77.92	78.52	79.44	79.25	79.74
ViT-B	50	224 × 224	77.46	77.35	78.02	78.90	78.91	79.66
ViT-L	50	224 × 224	79.51	79.36	79.88	80.67	80.61	81.86
SwinT-B	50	224 × 224	78.57	78.55	79.17	80.81	80.74	81.61
SwinT-L	50	224 × 224	79.58	79.53	80.11	81.77	81.72	82.52
Swinv2-B	50	192 × 192	77.36	77.33	77.98	80.30	80.27	80.92
Swinv2-L	50	192 × 192	78.31	78.30	78.89	80.22	80.23	80.86
DeiT-S	50	224 × 224	72.62	72.63	73.39	75.28	75.28	75.98
DeiT-B	50	224 × 224	75.91	75.91	76.65	77.41	77.38	78.07
DeiTv2-B	50	224 × 224	75.52	75.41	76.03	78.05	78.06	78.70
DeiTv2-L	50	224 × 224	77.54	77.43	78.14	79.07	78.96	79.58
Twins-B	50	224 × 224	75.82	75.77	76.44	77.61	77.53	78.11
Twins-L	50	224 × 224	76.04	75.93	76.60	78.13	78.00	78.65
Cait-S	50	224 × 224	76.41	76.40	77.08	77.87	77.76	78.46
CSWin-L [42]	50	224 × 224	79.90	–	–	–	–	–
VOLO-D5 [42]	50	224 × 224	79.51	–	–	–	–	–
Inception V3 [43]	50	224 × 224	–	–	–	76.17	–	–
WRN [44]	50	224 × 224	–	–	–	79.76	–	–
Swin-DR [7]	50	224 × 224	81.07	80.98	81.48	**82.77**	82.41	83.12
**Swin-ACST**	50	224 × 224	**82.28**	**82.21**	**82.76**	82.64	**82.60**	**83.39**

**Table 2:** Ablation study of different module combinations in Swin-ACST on FoodX-251 and UEC Food-256 datasets. **S**: Swin-DR, **C**: CEAG, **A**: AGRA, **SG**: SGLR-Mixer, **T**: TSCA-Classifier.

Model Variant	S	C	A	SG	T	Acc. (%)	Pre. (%)	F1. (%)	Acc. (%)	Pre. (%)	F1. (%)
	Modules					FoodX-251			UEC Food-256		
S	✓	✗	✗	✗	✗	81.07	80.98	81.48	82.15	82.11	82.89
S + C	✓	✓	✗	✗	✗	81.41	81.52	82.09	82.36	82.28	83.16
S + A	✓	✗	✓	✗	✗	81.38	81.26	81.93	82.29	81.95	83.03
S + C + A	✓	✓	✓	✗	✗	81.67	81.47	82.12	82.48	81.99	83.13
S + C + A + SG	✓	✓	✓	✓	✗	81.97	81.64	82.44	82.54	82.17	83.22
S + C + A + SG + T (Ours)	✓	✓	✓	✓	✓	82.28	82.21	82.76	82.64	82.60	83.39

## Data Availability

The original data presented in the study are openly available. UEC FOOD-256 is available at http://foodcam.mobi/dataset.html (accessed on 11 December 2024). FoodX-251 is available at https://www.selectdataset.com/dataset/439a25421e7c974dd41dc26a3e40e42c (accessed on 11 December 2024). All datasets can be used for academic purposes in accordance with their respective terms of use or open-source licenses.
